# Acute and subacute toxicity tests of goat bile in BALB/c mice

**DOI:** 10.14202/vetworld.2020.515-520

**Published:** 2020-03-20

**Authors:** Heny Arwati, Windya T. Hapsari, Kartika A. Wardhani, Kholida N. Aini, Ramadhani R. Bahalwan, Puspa Wardhani, Willy Sandhika

**Affiliations:** 1 Department of Parasitology, Faculty of Medicine, Universitas Airlangga, Surabaya, Indonesia; 2 HVA Hospital, Pare, Kediri, Indonesia; 3 Master Program of Immunology, Postgraduate School, Universitas Airlangga, Surabaya, Indonesia; 4 Department of Pharmacology, Faculty of Medicine, Universitas Airlangga, Surabaya, Indonesia; 5 Department of Clinical Pathology, Faculty of Medicine, Universitas Airlangga, Surabaya, Indonesia; 6 Dr. Soetomo Hospital, Surabaya, Indonesia; 7 Department of Anatomic Pathology, Faculty of Medicine, Universitas Airlangga, Surabaya, Indonesia

**Keywords:** BALB/c mice, goat bile, Indonesia, toxicity

## Abstract

**Aim:**

The aim of this study was to investigate the toxicity of goat bile in BALB/c mice since some Indonesian people consume raw goat gallbladder to treat malaria and increase stamina.

**Materials and Methods:**

Acute toxicity test was done in six groups of BALB/c mice using 100%, 50%, 25%, 12.5%, and 6.75% of goat bile and negative control. The death of mice was observed within 14 days. In the subacute toxicity test, the body weight and hematology parameters on day 0 and day 4 post-treatment were evaluated. The mice were closely observed for 28 days before plasma collection for the blood biochemistry evaluation.

**Results:**

Mild diarrhea was observed in acute and subacute toxicity tests. No death of mice was observed in acute test. Goat bile did not inhibit the increase of the body weight of mice. A slight reduction in hemoglobin and hematocrit levels in mice treated with 25% and 50% goat bile, however, remained normal in mice treated with 100% goat bile. The red and white blood cell count were not affected. Liver and kidney functions were not affected by goat bile treatment as revealed by the plasma level of aspartate aminotransferase and alanine aminotransferase, blood urea nitrogen, and creatinine, which remained in the normal range.

**Conclusion:**

Goat bile treatment in BALB/c mice caused mild toxicity in mice. Hydrophobic bile acids may cause the toxicity of goat bile in mice; therefore, it is recommended that goat bile consumption not to be taken oftenly to avoid its harmful effect.

## Introduction

Malaria control in Indonesia is based on the use of antimalarial drugs such as artemisinin-based combination therapy which is recommended by the WHO [1], however, some people of Indonesia traditionally consume an intact goat gallbladder to treat malaria because is believed not to be bitten by mosquitoes and to increase their stamina [2]. Goat gallbladder is a part of the goat’s body that is not consumed due to its bitter taste. Gallbladder is a small pouch where bile is stored. Bile is a unique digestive liquid that is continually secreted from hepatocytes and involves in biliary system in human, most animals including mammals and reptiles. This system involves liver, gallbladder, the hepatic, and bile ducts. Bile contains bile acids, which are critical for digestion and absorption of fats and fat-soluble vitamins in the small intestine [3,4].

The most components of bile are steroidal detergent-like molecules and the unesterified cholesterol, phosphatidylcholines, and bilirubin. Bile from various animals and some bile components combined with herbal medicines and other materials have been used for centuries as components of traditional Chinese medicine (TCM) to treat chronic and acute infectious and non-infectious diseases including malaria. Based on the information on biliary chemistry such as specific bile salts, bile pigment bilirubin and its glucuronides, the minor components of bile such as Vitamins A, D, E, K, as well as melatonin (N-acetyl-5-methoxytryptamine), animal biles have been reported improve the liver function, dissolving gallstones, inhibiting bacterial and viral multiplication, as well as exhibiting anti-inflammatory, antipyretic, antioxidant, sedative, anticonvulsive, antiallergic, anti-congestive, antidiabetic, and antispasmodic effects. [5,6]. Goat bile is more rarely used in traditional Chinese medicine. Goat bile was used therapeutically in China as it was believed to be effective in treating optic atrophy, acute hemorrhagic conjunctivitis, and various infectious skin diseases, as documented in Chinese materia medica [5].

The aim of this study was to investigate the toxicity of goat bile in BALB/c mice since some Indonesian people consume raw goat gallbladder to treat malaria and increase stamina.

## Materials and Methods

### Ethical approval

The proposal of this research has been reviewed by the Ethics Committee of Faculty of Medicine, Universitas Airlangga, as described on the Ethical Clearance No. 195/EC/KEPK/FKUA/2018.

### Preparation of goat bile

Goat gallbladders were bought from the local animal slaughterhouse, in Surabaya, East Java Province. Java strain of goat was chosen for this experiment as the most consumed in Surabaya. Goat gallbladders were isolated from four healthy male goats for each test. Gallbladders were sprayed with 70% alcohol, before removal of the bile by syringe, transferred and pooled to a clean tube then diluted with distilled water to prepare 100%, 50%, 25%, 12.5%, and 6.25% goat bile, and stored at 4°C during the experiment.

### Acute toxicity test

The goat bile was tested in healthy male BALB/c mice aged 6-8 weeks and weighing 20-30 g. Mice were divided randomly into six groups of five mice per group for the administration of the following concentrations of goat bile, 100%, 50%, 25%, 12.5%, and 6.25% in distilled water, respectively, and negative control group. The mice were starved for 4 h before the experiment began and only gave water *ad libitum*. The mice in each group were given 0.5 ml/25 g body weight of each concentration using gavage [7]. The mice in negative control group were given 0.5 mL of distilled water. The mice were observed continuously for 1 h and 24 h and thereafter daily for 14 days [7]. The observation was done for any manifestation of toxicity including changes in skin and fur, eyes and mucous membranes, respiratory and digestive distress and urine output, behavior pattern, coma, and death.

### Subacute toxicity test

Twenty mice were grouped into four groups. Groups 1-3 were administered orally for 4 days with 100% (GB100), 50% (GB50), and 25% (GB25) goat bile, each mouse received 0.5 ml/25 g body weight of goat bile. Group 4 was given 0.5 ml of distilled water four each mouse. The body weight of each mouse was recorded on days 0 and 4, followed by the examination of the blood biochemical, including hemoglobin (HGB), hematocrit (HCT), and red blood cell (RBC) and white blood cell (WBC) counts [7]. The mice were then closely observed for 28 days [8] before plasma collection for the blood biochemistry evaluation.

### Blood biochemistry evaluation

On day 28, mice were sacrificed, and blood was collected by cardiac puncture and transferred to EDTA Vacutainer tubes. Blood was analyzed for the function of liver including aspartate aminotransferase (AST), alanine aminotransferase (ALT), and function of kidney including blood urea nitrogen (BUN) and creatinine using automatic hematology analyzer. Biochemistry and hematological analyses were performed in the Department of Clinical Pathology, Dr. Soetomo Hospital, Faculty of Medicine, Universitas Airlangga.

### Statistical analysis

The data of blood biochemistry were analyzed using one-way analysis of variance (ANOVA), if variances of the groups were assumed to be equal. When ANOVA showed statistical significance, Bonferroni or Games-Howell (*post hoc*) multiple tests were used to determine the significance of differences among groups. If variances of the groups were not assumed to be equal, Mann–Whitney U-test was used to determine the significance of the group differences. Two-tailed paired t-test was used to compare the mean body weight and hematology values before and after treatment. The result was considered statistically significant at 95% confidence level and p<0.05. The data were entered in Microsoft Excel spreadsheet, exported and analyzed using SPSS version 20 (IBM Corp., NY, USA).

## Results

### Acute toxicity test

Observation of the acute toxicity of goat bile in non-infected mice showed that mice treated with the lowest to the highest concentration of goat bile did not show any changes in skin and fur, eyes and mucous membranes, respiratory and digestive distress, behavior pattern, and coma. Mild diarrhea was observed only in mice treated with GB100 within 2 days and recovered afterward, however, no decrease in urine output. This result might indicate mild intestinal toxicity but not in urinary tract. No death of mice was observed in this test. Hence, all concentrations of goat bile used in the test were safe for *in vivo* study in mice at least for a 4-day treatment.

### Subacute toxicity test

#### Physical observation of mice

**Figure-1:**
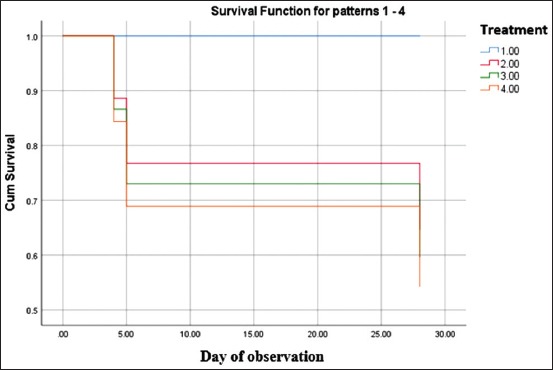
Survival pattern of mice in goat bile-treated groups and negative control. All mice (5) of negative control group survived (100%) within 28 days of observation. Two mice of GB100-, GB50-, and GB25-treated group died on days 4, 5 and 28, respectively, and percentages survival of those three groups were total 60%. Blue line, treatment 1: Negative control; Red line, treatment 2: GB100-treated mice; Green line, treatment 3: GB50-treated mice; Orange line, treatment 4: GB25-treated mice. Statistical analysis: Cox regression test.

The concentrations of goat bile used in this test were based on the results of acute toxicity test. Mice looked normal in their mobility and food and water consumption. Mild diarrhea was also observed within the first 2 days of treatment in GB100-treated group, then showed normal afterward. However, two mice out of five died after underwent this symptom on day 4 post-treatment. Two mice in GB50 group died on day 5 and two mice of GB25 group died on day 28 without any diarrhea symptom. This result indicated that the mice treated with the higher concentration of GB causing the earlier death of mice. The control group remained normal. The percentages survival of mice in GB25-, GB50-, and GB100-treated groups in Cox regression test were all 60%, therefore, there was no significant difference in survival rate in all GB-treated groups (p=0.449). All mice in negative control were 100% survived. The curve of percentages survival is presented in Figure-1.

#### Body weight

The data of body weights of goat bile-treated mice are presented in Table-1. The body weights of mice in goat bile-treated groups were constant or slightly increased not significantly from day 0 to day 4 as well as in negative control (p>0.05). Goat bile did not inhibit the increased body weight of mice within 4 days as compared with the control group.

**Table-1:** Subacute toxicity effect of goat bile on body weight and hematology parameters in BALB/c mice.

Group of mice	Parameters	Day 0	Day 4	p-value
GB25	BW	19.00±1.00	19.00±0.71	1.000
	HGB	13.54±1.64	8.86 ±4.62	0.108
	HCT	47.98±6.14	30.98±17.02	0.050
	RBC	9.96±0.65	8.12±58	0.016*
	WBC	5.64±1.35	4.90±1.32	0.879
GB50	BW	19.80±2.49	20.67±1.53	0.199
	HGB	15.02±1.73	10.2±0.10	0.001*
	HCT	53.76±1.17	20.17±14.18	0.057
	RBC	10.71±0.29	6.38±0.79	0.100
	WBC	8.43±0.25	8.45 ±1.68	0.821
GB100	BW	20.6±2.41	21.67±2.89	0.423
	HGB	12.97±0.06	17.27±0.31	0.059
	HCT	50.56±3.21	59.07±1.29	0.053
	RBC	10.10±0.70	11.45±0.21	0.952
	WBC	5.00±1.13	8.76±0.21	0.136
GBNeg	BW	19.75±1.71	21.25±1.00	0.103
	HGB	15.03±1.29	12.48±1.54	0.001*
	HCT	51.75±4.92	47.10±2.03	0.404
	RBC	9.82±1.30	8.79±0.79	0.530
	WBC	5.48±2.10	6.55±3.16	0.118

Gb25=Goat bile 25%, GB50=Goat bile 50%, GB100=Goat bile 100%, GBNeg=Negative control (sterile water), BW=Body weight, HGB=Hemoglobin, HCT=Hematocrit, RBC=Red blood cell, WBC=White blood cell.

* Significantly different between parameters on day 0 and day 4

#### Hematology evaluation

Hematology data of mice treated with goat bile compared with untreated mice are presented in Table-1. Statistical analysis using paired sample t-test on the difference of the hematological parameters on day 0 and day 4 showed no significant HGB reduction (p>0.05) in mice treated with GB25 and normal mice. Only HGB in mice treated with GB50 reduced significantly on day 4 (p=0.001). Similarly, RBC counts reduced significantly in mice treated with GB25 (p=0.016), but not significantly in GB50-treated mice and negative control. HCT levels were also increased not significantly in mice treated with GB25, GB50, and negative control. The WBC counts were not different significantly in all mice. Interestingly, no significant increase in HGB and HCT levels, RBC and WBC counts were seen in mice treated with GB100 (p>0.05).

#### Blood biochemistry evaluation

The plasma level of AST, ALT, BUN, and creatinine is shown in Table-2. Data were analyzed using two independent sample t-test and independent sample t-test showed that only the level of AST of mice treated with GB100 different significantly with that of untreated mice (p=0.006). Similar results were seen in plasma level of creatinine of mice treated with GB100 different significantly with untreated mice where p=0.029. Plasma level of AST increased along with the increase of the concentration of GB. The higher concentration of GB caused a higher concentration of plasma level of AST. This result indicated that plasma level of AST was concentration-dependent. On the other hand, plasma level of ALT decreased along with the increase of goat bile concentration. However, the plasma level of AST, ALT, BUN, and creatinine of goat bile-treated mice remained normal compared with those of control group. There were no differences in the plasma level of AST, ALT, BUN, and creatinine of mice treated with GB25 and GB50 compared with those of negative control (p>0.05).

**Table-2:** Blood biochemistry in mice treated with goat bile compared with negative control.

Group of mice	Parameter	Plasma level	p-value
GB25	AST	99.25±29.769	0.315
	ALT	75.33±77.732	0.109
	BUN	17±2.160	0.918
	Creatinine	0.12±0.079	0.686
GB50	AST	108.00±23.516	0.183
	ALT	47.67±10.116	0.238
	BUN	18.33±4.041	0.575
	Creatinine	0.115±0.007	0.057
GB100	AST	118.67±10.116	0.006*
	ALT	31.667±5.033	0.567
	BUN	17.33±3.214	0.931
	Creatinine	0.06±0.04	0.029*
GBNeg	AST	94.50±5.000	
	ALT	44.25±4.113	
	BUN	17.67±0.289	
	Creatinine	0.157±0.015	

GB25=Goat bile 25%, GB50=Goat bile 50%, GB100=Goat bile 100%, GBNeg=Negative control (sterile water), AST=Aspartate aminotransferase, ALT=Alanine aminotransferase, BUN=Blood urea nitrogen.

* Significantly different with GBNeg

## Discussion

The acute and subacute toxicity tests resulted in mild toxicity of goat bile due to mild diarrhea within 2 days post-treatment. On day 3-4 post-treatment, such symptom was disappeared. Mild diarrhea found in mice treated with GB100 was similar to the people in India who consumed toxic fish gallbladder but with severe clinical manifestations include abdominal pain and watery diarrhea several hours later, however, the effect of fish gallbladder consumption is more severe because it was followed by the manifestations of oliguria and renal failure [9]. Several toxicity cases after consuming fish gallbladders have been reported in India [9,10] and Cambodia [11]. Fish gallbladder is believed to improve eye vision and treat rheumatism. The bile of grass carp fish contains highly virulent toxin. One of the toxic components is water-soluble sodium cyprinol sulfate, which can lead to multiple organs dysfunctions, but most of fish gallbladder contain ciguatoxin. The poison leads to acute renal failure, acute liver injury, and therefore increasing mortality [9-11]. Information on the component of goat bile is very little. The component of goat bile has been reported was similar to sheep bile as the absolute amounts of cholesterol, phospholipids, free fatty acids, carbohydrates and dry matter secreted by goat are much lower than that by sheep [12]. However, there is no description on the toxin content of goat bile as well as no report on the goat bile poisoning. Consuming the goat gallbladder is constrained by its size and component of bile, which sometimes is toxic. The smaller size of goat gallbladder should be chosen by Indonesian people to be easier to swallow [13].

Goat bile did not inhibit the increase of body weight of mice within 4 days of observation as shown by the increase of body weight normally (Table-1). Hematology parameters remained normal [14], even a slight reduction in HGB and HCT levels in mice treated with GB25 and GB50 was observed, however, remained normal in mice treated with GB100. Other parameters, RBC and WBC counts were not affected. Decreasing of RBC count due to suicidal erythrocytes death (eryptosis) which is triggered by bile acid [15] did not occur in this experiment.

Liver enzymes such as AST and ALT are the major characteristic of liver function. Elevation of those enzymes in plasma indicated liver dysfunction. Only mice treated with GB100 showed reducing the plasma level of AST and ALT insignificantly (p>0.05) when compared with the control group. Lower concentration of goat bile caused no significant change in liver function as revealed by the plasma level of AST and ALT, which remained in normal range compared with reference of normal mice of 55-352 IU/L and 41-131 IU/L [14]. These results indicated that goat bile treatment in various concentrations did not affect the function of liver. The ethnomedical data prove that the use of animal biles improves the stamina and salutary in improving liver function [5]. Animal bile has been known to treat liver diseases [6]. In addition, no elevation level of BUN and creatinine indicated that the kidney function was not affected by goat bile treatment. This condition exactly different with fish toxic gallbladder consumption in India which caused acute renal failure [9].

The limitation of this research was that the toxicity assays were done using the whole bile without any further characterization of the component as Indonesian people do so because this research was only proving whether goat bile toxic or not. The difficulties in this research were in obtaining sufficient volume of goat bile. The biles should be pooled from several goat gallbladders to meet the adequacy volume, even though the component of each bile may different. One of the animal’s biles which have been used medically is bear bile that effective in treating a number of infectious and non-infectious diseases, such as ascariasis and oxyuriasis in children as well as blood retention syndrome as well as liver diseases [5,6]. The composition of bear bile was not constant, it changed by a variety of factors such as existing style, species, physical state, and season [6]. Similarly, many existing strains of goat in Indonesia, the composition of bile may also different. The variation of bile composition is crucial and has great importance to identify the quality of bile. The compounds of bile may provide scientific basis to further investigate their pharmacological actions and mechanisms [6].

The clinical uses of goat bile reveal some advantages. Although some animal biles exhibit generic effects, a number of bile possess advantages in specific therapeutic indications. Clinical efficacy of the medicinal uses of the different animal biles is based on their chemical components. In TCM, goat and sheep biles were considered to have similar therapeutic effects. They were believed to be effective in treating optic atrophy, in ameliorating various infectious skin diseases and also constipation. Goat bile was also used to treat temporary blindness and eye injury from foreign bodies. The volume of bile per gallbladder of goat was lower (±8.6 mL) as compared to that of sheep (±18.6 mL). Goat bile contains cholesterol, phospholipid, free fatty acids, carbohydrates, and dry matter. Goat salt bile is composed of glycocholate, glycodeoxycholate, taurocholate, taurochenodeoxycholate, and taurodeoxycholate. Bile pigment in goat bile consists of bilirubin monoglucuronide[5,12].

Based on the results, mild toxicity of goat bile caused the death of two mice who underwent mild diarrhea, but the other three mice in the same group survived. The different self-limiting in responding to goat bile treatment may cause the different mortality and survival of mice, while the toxicity of bile caused by the complexity of its component. Bile acids may play a dual role due to their amphipathic property [16,17] with a hydrophobic side and a hydrophilic side [18]. Deoxycholic acid (DCA) as hydrophobic bile acid increases lipid polarity and fluidity [19], leading to damage of the cell membrane [20]. Ursodeoxycholic acid (UDCA) and tauroursodeoxycholic acid (TUDCA) as hydrophilic bile acids act as emollients with softening, soothing, cleansing, and mild antiseptic actions [5] are able to reverse the effects [19] and protect against toxicity of hydrophobic bile acids [21]. On the one side, bile acids are toxic, but on the other side act beneficially. Unique properties of bile acids have been considered use in drug delivery system and as therapeutic agents [22] in cancer diseases [23,24], malaria [25], and enteric infection [22,26].

More than a decade ago, the investigation on the toxicity of bile has been performed in rats. The death of rats following intravenous injection of ox bile was ascribed due to the toxicity of bile. The view gained ground that the death was not due to the toxicity of bile, but to impurities in the bile used which gave rise to the formation of capillary thrombi in vital nerve centers. Further proved when the filtered bile was given, the animal survived without manifesting any important nervous symptoms [27]. In fact, TUDCA and UDCA may have therapeutic role in neurodegenerative disease [28]. In this current research, no nervous symptoms were observed on the death of mice. The death of mice following oral treatment of goat bile tends to be due to hydrophobic bile acid toxicity rather than impurities of bile.

## Conclusion

Goat bile treatment in BALB/c mice caused mild toxicity, as shown by mild diarrhea in two mice treated with 100% goat bile. The toxicity of goat bile in mice may cause by hydrophobic bile acids; therefore, it is recommended that goat bile consumption not to be taken oftenly to avoid its harmful effect.

## Authors’ Contributions

HA: Research project leader, coordinator, and designed the research, analyzed data, drafted manuscript, and performed subacute toxicity test. WTH: Acute toxicity test. KAW and KNA: Sacrificed the mice, collected blood, and organized the data. RRB: Originator of research ideas and consultant. PW: Hematology and blood biochemistry analysis, WS: Clinical symptoms observer and consultant. All authors read and approved the final manuscript.
